# Genetic characterization of measles virus in the Philippines, 2008–2011

**DOI:** 10.1186/s13104-015-1201-1

**Published:** 2015-06-03

**Authors:** Rex Centeno, Naoko Fuji, Michiko Okamoto, Clyde Dapat, Mariko Saito, Amado Tandoc, Socorro Lupisan, Hitoshi Oshitani

**Affiliations:** Research Institute for Tropical Medicine (RITM), Alabang, Muntinlupa City, Philippines; Department of Virology, Graduate School of Medicine, Tohoku University, 2-1 Seiryo-machi, Aoba-ku, Sendai, Miyagi Prefecture 980-8575 Japan; Tohoku-RITM Collaborating Research Center for Emerging and Reemerging Infectious Diseases, Alabang, Muntinlupa City, Philippines

**Keywords:** Measles, Genotyping, Molecular epidemiology, Molecular clock analysis

## Abstract

**Background:**

Large outbreaks of measles occurred in the Philippines in 2010 and 2011. Genetic analysis was performed to identify the genotype of measles virus (MeV) that was responsible for the large outbreaks.

**Methods:**

A total of 114 representative MeVs that were detected in the Philippines from 2008 to 2011 were analyzed by sequencing the C-terminal region of nucleocapsid (N) gene and partial hemagglutinin (H) gene and by inferring the phylogenetic trees.

**Results:**

Genetic analysis showed that genotype D9 was the predominant circulating strain during the 4-year study period. Genotype D9 was detected in 23 samples (92%) by N gene sequencing and 93 samples (94%) by H gene analysis. Sporadic cases of genotype G3 MeV were identified in 2 samples (8%) by N gene sequencing and 6 samples (6%) by H gene analysis. Genotype G3 MeV was detected mainly in Panay Island in 2009 and 2010. Molecular clock analysis of N gene showed that the recent genotype D9 viruses that caused the big outbreaks in 2010 and 2011 diverged from a common ancestor in 2005 in one of the neighboring Southeast Asian countries, where D9 was endemic. These big outbreaks of measles resulted in a spillover and were associated with genotype D9 MeV importation to Japan and the USA.

**Conclusion:**

Genotype D9 MeV became endemic and caused two big outbreaks in the Philippines in 2010 and 2011. Genotype G3 MeV was detected sporadically with limited geographic distribution. This study highlights the importance of genetic analysis not only in helping with the assessment of measles elimination program in the country but also in elucidating the transmission dynamics of measles virus.

**Electronic supplementary material:**

The online version of this article (doi:10.1186/s13104-015-1201-1) contains supplementary material, which is available to authorized users.

## Background

The measles virus (MeV) is a highly contagious pathogen, which causes a disease in humans characterized by prodromal symptoms that include high fever, cough, runny nose, conjunctivitis, and tiny white spots on the inside of the mouth before the onset of rash [1]. MeV belongs to order *Mononegavirales*, family *Paramyxoviridae*, genus *Morbillivirus* and contains nonsegmented single-stranded RNA genome of negative polarity that is 16 kb in length [2]. The genome codes for six viral proteins namely; hemagglutinin (H) and fusion (F) glycoproteins, matrix (M) protein, nucleocapsid (N) protein, phosphoprotein (P), and large polymerase (L) protein [2]. The C-terminal hypervariable region of the N gene (N-450) or the full length H gene is used in the genetic characterization of MeV and monitoring of measles control programs [3]. To standardize the nomenclature of MeV, the World Health Organization (WHO) established a systematic classification of the genetic characteristics of wild-type viruses [4].

Vaccination remains the first line of defense for measles virus infection. Before the introduction of measles vaccine in the 1960s, the disease was responsible for millions of deaths per year worldwide [1]. In the Philippines, the monovalent measles vaccine was introduced in 1983 and the trivalent vaccine composed of measles, mumps, rubella (MMR) was introduced in 1990 [5]. Supplementary immunization activities (SIAs) were conducted in the country in 1998 (coverage 85%); 2004 (95%); 2007 (95%), and 2011 (84%) [5]. Before the introduction of nationwide mass measles vaccination campaign in the Philippines, the estimated deaths among children caused by measles was about 6,000 [6].

To measure the country’s progress towards measles elimination and monitor the effectiveness of mass vaccination campaigns, regular surveillance is carried out by the Department of Health. Laboratory confirmation of suspected measles cases is conducted at the Research Institute for Tropical Medicine (RITM) in Metro Manila by detecting measles-specific IgM antibody [7]. However, genetic analysis is not performed routinely so there is limited information on the current circulating strains. Previous reports showed that the number of measles was reduced in 2005, after a nationwide mass vaccination campaign in 2004 [6], and genetic analysis of samples collected between 2000 and early 2008 showed that the circulation of an endemic D3 genotype was interrupted and was replaced by an imported D9 genotype in 2007 [8]. However, measles outbreak were reported in the Philippines in 2010 and 2011 [5]. This study was conducted to determine if a new genotype was responsible for the measles outbreaks in 2010 and 2011. This study also aimed to characterize the molecular evolution and geographic distribution of genotypes of measles virus in the Philippines.

## Results

### Epidemiology of measles in the Philippines

**Figure 1:**
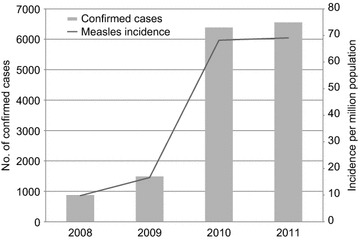
The number of reported cases and incidence of measles in the Philippines, 2008–2011. *Gray bars* represent the number of confirmed cases of measles and *black line* represents the incidence rate of measles per one million population. The data was obtained from the WHO report on the regional and country profile of measles elimination in the Philippines [5].

The number of reported confirmed cases and incidence of measles in the Philippines from 2008 to 2011 were obtained from the WHO regional and country profiles of measles elimination report (Figure 1) [5]. Significant increase of measles cases was reported in the Philippines in 2010 and 2011, which showed a 7-fold increase in the incidence rate of 69.1 per 1 million population in 2011, when compared with the data in 2008. The number of deaths attributed to measles also increased from 8 deaths in 2008 to 28 deaths in 2011 [5].

During the study period, a total of 7,437 IgM ELISA-positive samples were collected at RITM and of these, 565 samples were selected and tested for PCR assay using primers specific for the N and H genes (Table 1). Results showed that of the 114 samples that were tested positive by PCR, 25 and 99 samples were positive for N and H genes respectively.

**Table 1:** Yearly distribution of IgM ELISA and PCR positive samples and genotypes of measles virus in the Philippines

	2008^a^	2009	2010	2011	Total
IgM ELISA positive	120	698	2,973	3,646	7,437
Number of PCR samples tested	16	65	228	256	565
H gene PCR positive	3	13	53	30	99
N gene PCR positive	2	5	9	9	25
D9 genotype	2	9	51	29	91
G3 genotype	1	3	2	0	6
Undetermined genotype	0	1	0	1	2

^a^Data from July 2008.

### Phylogenetic analysis of MeV in the Philippines

**Figure 2:**
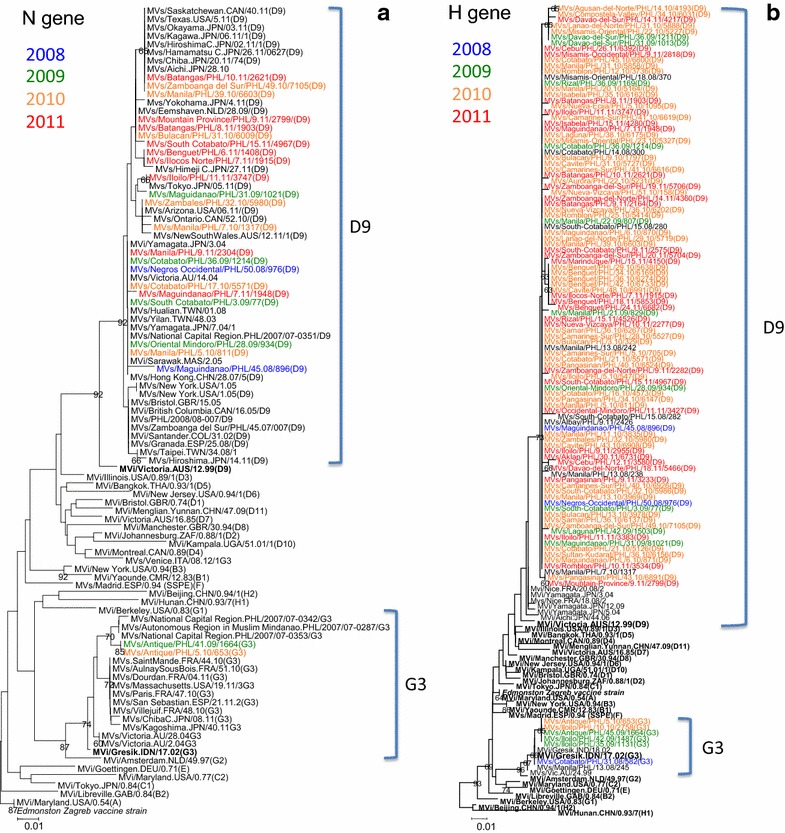
Phylogenetic trees based on the C-terminal region of N gene (**a**) and partial H gene (**b**) sequences of MeVs in the Philippines, 2008–2011. The tree was constructed using the NJ method with bootstrap of 1,000 replications as implemented in MEGA 5.0 software. WHO reference strains are *boldfaced*. Samples that were detected in 2008 are indicated by *blue* font color; 2009 (*green*); 2010 (*orange*); and 2011 (*red*). The *scale bar* represents nucleotide substitution per site.

Phylogenetic analysis of the 450-nt C-terminal hypervariable region of N gene showed that the 2008–2011 MeVs in the Philippines belonged to two genotypes, D9 and G3 (Figure 2a). Of the 25 representative N gene sequences analyzed, 23 samples (92%) clustered with the genotype D9 reference strain (92% bootstrap value) and 2 samples (8%) clustered with the genotype G3 reference strain (87% bootstrap value). Similar clustering was observed using the Bayesian Markov chain Monte Carlo (MCMC) method with posterior probability values of 1.0 and 0.99 for genotypes D9 and G3 respectively (Additional file 1: Figure S1). The two cases of genotype G3 in the Philippines formed a separate group and did not cluster with MeVs from other countries.

Pairwise distance analysis of N gene showed high sequence similarity between Philippine strains and reference strains from other countries (Additional file 2: Table S1). Three Philippine strains (Batangas/PHL/10.11, Zamboanga del Sur/PHL/49.10, Manila/PHL/39.10) exhibited 100% sequence similarity with 1 strain in Canada (Saskatchewan.CAN/40.11), 1 strain in the USA (Texas.USA/5.11), and 6 strains in Japan (2010–2011). Another three Philippine strains (Mountain Province/PHL/9.11, Batangas/PHL/8.11, Bulacan/PHL/31.10) showed high sequence similarity with MeV from the Netherlands (Eemshaven/NLD/28.09). One strain from central Philippines (Iloilo/PHL/11.11) showed high sequence identity with a strain from Japan (Tokyo.JPN/05.11). One strain from Luzon (Zambales/PHL/32.10) showed 100% sequence similarity with a strain from the USA (Arizona.USA/06.11). Eight Philippine strains showed high sequence similarity with older MeV viruses from Taiwan (2003), Japan (2004), Australia (2004), and Malaysia (2005). Two Philippine strains showed high sequence similarity with MeVs from Columbia (2002), UK (2005), Canada (2005), and Spain (2008). In 2010 and 2011, a total of 4 cases of genotype D9 virus infection in Japan and 6 cases in the USA showed clear epidemiological link to the MeV infections in the Philippines [9–11].

Phylogenetic analysis of MeVs that were detected in the Philippines from 2008 to 2011 showed that the partial H gene phylogeny was congruent with the N gene phylogeny in the NJ method (Figure 2b) and MCMC method (Additional file 3: Figure S2). Of the 99 representative H gene sequences analyzed, 93 samples (94%) grouped together with the genotype D9 reference strain (73% bootstrap value) and 6 samples (6%) grouped together with the genotype G3 reference strain (96% bootstrap value).

Analysis of the partial H gene sequence of the 6 cases of genotype G3 MeVs in the Philippines showed similar clustering with the N gene phylogeny (Figure 2b). Within the G3 cluster, one sample (MVs/Cotabato/PHL/31.08/582) grouped with the sample that was detected in Manila in early 2008 and the remaining five samples that were detected in 2009 and 2010 formed another separate group.

**Figure 3:**
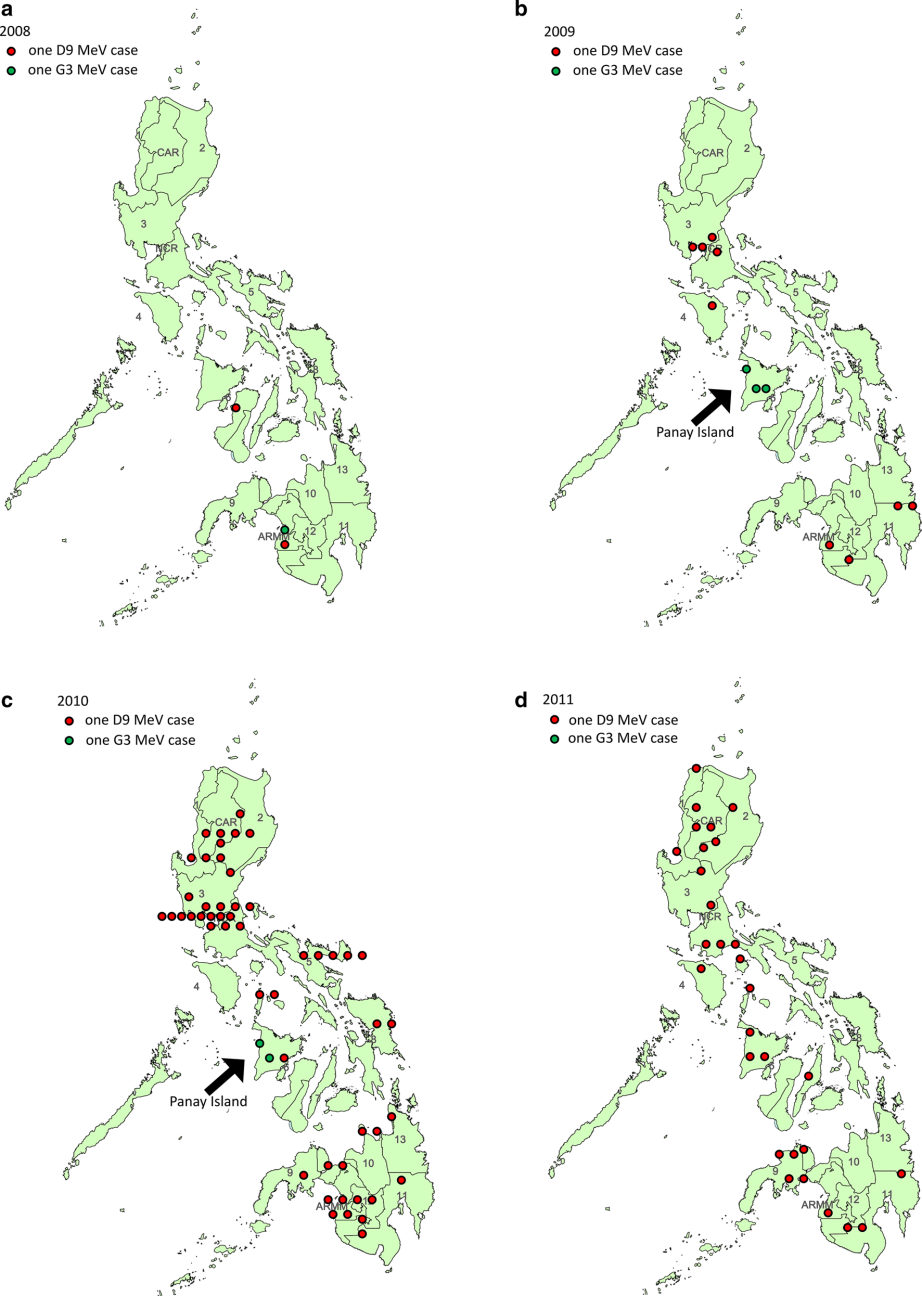
Geographic distribution of genotypes D9 and G3 in the Philippines. Distribution of genotypes D9 (*red circle*) and G3 (*green circle*) in 2008 (**a**), 2009 (**b**), 2010 (**c**), and 2011 (**d**). *One circle* represents one case of MeV infection. *Numbers* represent the different administrative regions in the country. *CAR* Cordillera Autonomous Region, *NCR* National Capital Region, *ARMM* Autonomous Region in Muslim Mindanao.

Mapping of the geographic location of measles cases in the Philippines according to genotype showed that the cases of genotype D9 MeVs were detected in all 16 regions in the country (Figure 3a–d). One case of genotype G3 was detected in Mindanao in 2008 (Figure 3a) and the remaining five cases of G3 were detected mainly in the island of Panay (Region 6) in 2009 (Figure 3b) and in 2010 (Figure 3c). Both genotypes D9 and G3 were detected in Panay Island in 2010, however, only genotype D9 MeV continued to circulate in the following year and G3 MeVs were not detected in 2011. Some groups with identical sequences (e.g. MVs/Lanao-del-Norte/PHL/31.10/5888, MVs/Misamis-Oriental/PHL/22.10/5227, MVs/Davao-del-Sur/PHL/36.09/1211, MVs/Davao-del-Sur/PHL/31.09/1013) showed geographic clustering, while other groups with identical sequences (e.g. MVs/Cotabato/PHL/45.10/6800, MVs/Manila/PHL/31.10/5858, MVs/Romblon/PHL/12.10/3736) were detected in different islands within the country (Additional file 4: Table S2; Figure 3b, c).

### Molecular evolution of MeV in the Philippines

The molecular evolution of the N gene of the MeVs in the Philippines was analyzed using MCMC method. Results showed that the mean nucleotide substitution rate of N gene is 2.78 × 10^−3^ [95% highest posterior density (HPD) interval: 7.2 × 10^−4^ to 4.96 × 10^−3^] substitutions per site per year, which was estimated using the uncorrelated lognormal relaxed clock-constant population growth model combination that was determined as the best-fitting model (Table 2). These estimates are similar to previously reported mean mutation rates of MeV in Spain: 3.0 × 10^−3^ substitutions/site/year for genotype C2 MeV from 1992 to 1993; 5.0 × 10^−4^ substitutions/site/year for genotype D6 from 1993 to 1996 [12]; and 2.66 × 10^−3^ substitutions/site/year for genotype B3 MeV in 2006 [13]. Similar estimate was also reported for genotype H1 MeV in China, from 1993 to 2012 with 1.65 × 10^−3^ substitutions/site/year [14]. Analysis of N gene sequences available in GenBank showed similar mutation rate of 8.69 × 10^−4^ substitutions/site/year [15]. The mean time to the most recent common ancestor (tMRCA) for genotype D9 MeVs in the Philippines is August 2005 [95% HPD: September 2001 to November 2007] and the tMRCA for genotype G3 is May 2008 [95% HPD: September 2007 to August 2008] (Table 2).

**Table 2:** The estimated substitution rates and the dates of most recent common ancestor by four different models

Molecular clock	Demographic model	Substitution rate^a^, ×10^−3^ (95% HPD)	tMRCA, D9 (95% HPD)	tMRCA, G3 (95% HPD)
Strict	Constant	2.54 (0.96–4.35)	2005 September 20 (2002 May to 2007 Nov)	2008 May 18 (2007 Oct to 2008 Aug)
Strict	Exponential growth	2.71 (1.10–4.47)	2006 January 12 (2003 Mar to 2007 Nov)	2008 May 29 (2007 Nov to 2008 Aug)
Lognormal relaxed	Constant	2.78 (0.72–4.96)	2005 August 3 (2001 Sept to 2007 Nov)	2008 May 15 (2007 Sept to 2008 Aug)
Lognormal relaxed	Exponential growth	3.37 (0.92–6.23)	2006 January 20 (2003 Jan to 2007 Nov)	2008 May 24 (2007 Oct to 2008 Aug)

*tMRCA* time to the most recent common ancestor, *HPD* highest posterior density interval.

^a^Substitutions/site/year.

MCC trees showed similar clustering of Philippine D9 and G3 genotypes by four different models, which is in agreement with the NJ distance method. Within D9 genotype, MeVs in the Philippines shared common ancestors with viruses from Colombia (MVi/Santander.COL/31.02) (group 1), Taiwan (MVs/Yilan/TWN/48.03) (group 2), Australia (MVs/Victoria.AU/14.04) (group 3), Malaysia (MVi/Sarawak.MAS/2.05) (group 4) (Additional file 1: Figure S1). Bayesian skyline plot analysis of H gene of genotype D9 MeV in the Philippines showed an increase in genetic diversity in 2010 and peaked in 2011 (Additional file 5: Figure S3).

## Discussion

Large outbreaks of measles were reported in the Philippines in 2010 and 2011 [5]. Genetic analysis showed that genotype D9 MeV was responsible for these large outbreaks in the country. From 2000 to 2004, genotype D3 was endemic in the Philippines [8]. The transmission of endemic genotype D3 MeVs was interrupted after a mass vaccination campaign in 2004 [6, 8]. Since 2005, genotype D3 has not been detected in countries belonging to the WHO Western Pacific Region [16]. In 2007, sporadic cases of genotypes D9 and G3 virus infections were detected in the country [8]. In this report, genotype D9 became the predominant strain in the Philippines in 2010 and 2011.

Genotype D9 was described first in Australia in 1999, which was an imported case from Indonesia [17]. Outbreaks of genotype D9 in Indonesia and Malaysia during the same period may suggest that D9 was endemic in these countries [17]. In 2002, a large measles outbreak occurred in Venezuela and spread to Colombia, where the index case developed measles after returning from a trip to Europe [18]. Sequence analysis revealed a close match with the imported MeV in Australia in 1999 [18]. In 2004, a measles outbreak occurred in a junior high school in Japan that was caused by genotype D9, which has not been detected previously in the country [19, 20]. Genetic analysis also showed a high sequence similarity with the 1999 MeV in Australia that was imported from Indonesia [19].

Before 2005, genotype D9 was not detected in the Philippines. D9 MeV infections in this period were associated with imported cases from Indonesia, East Timor, Malaysia, and Singapore where D9 was endemic [8, 16]. Notably, eight D9 MeVs that were collected from 2008 to 2011 exhibited high sequence similarity with MeVs in Malaysia in 2005. In addition, molecular clock analysis suggests that the recent genotype D9 viruses that caused the big outbreaks in 2010 and 2011 diverged from a common ancestor in 2005. These findings suggest that genotype D9 MeV was imported to the Philippines from neighboring countries around 2005.

Genotype D9 MeV was detected at an increasing rate in the Philippines since 2007. It has replaced the earlier endemic genotype G3 strain and became the predominant genotype. The increasing prevalence of D9 MeV has been detected in Taiwan, Thailand, and other neighboring countries [16, 21, 22]. In our previous study, the sequences of genotype D9 viruses that were detected in the Philippines in 2007 and 2008 shared high sequence similarity to those in Hong Kong (2007) and Taiwan (2003 and 2008) [8]. Of particular note, the genotype D9 cases in Taiwan in 2003 and 2008 were imported cases from Japan and the Philippines, respectively [23]. In this study, genotype D9 was the predominant strain in the country from July 2008 to 2011. Our data suggests that D9 MeV was responsible for the two big measles outbreak in 2010 and 2011.

The diversity of genotype D9 MeVs in the Philippines may be attributed to a combination of multiple importations and sustained local transmission. At least 4 importations of D9 genotype MeVs were observed in the Philippines, which shared common ancestors with MeVs from Colombia (MVi/Santander.COL/31.02) (group 1), Taiwan (MVs/Yilan/TWN/48.03) (group 2), Australia (MVs/Victoria.AU/14.04) (group 3), Malaysia (MVi/Sarawak.MAS/2.05) (group 4) (Additional file 1: Figure S1). During the big outbreaks in 2010 and 2011, group 3 and 4 viruses were cocirculating in the Philippines. Bayesian skyline plot analysis showed an increase in genetic diversity in 2010 and peaked in 2011 (Additional file 5: Figure S3), which suggests that endemic transmission of group 3 and 4 MeVs contributed to genetic diversity.

A limitation of this study is the use of partial H gene sequence for genotyping analysis. The WHO recommends the use of full H gene for genotyping [16]. However, only partial H gene sequences were obtained in this study because the samples used were from patients’ sera and not from virus isolates. Patients’ sera may contain low quality and insufficient amount of viral RNA. Despite this limitation, the H gene analysis was in agreement with N gene phylogeny and showed clustering of Philippines samples together with the D9 genotype reference strain and validated using two methods with a high bootstrap value of 92% for the NJ method and a posterior probability of 1.0 for the MCMC method. H gene analysis corroborated the findings of N gene phylogeny, which showed that the recent large outbreaks of measles in the Philippines were caused by D9 genotype MeV that has become established and sustained its transmission among susceptible populations. Sustained local transmission of D9 genotype MeV was supported by geographic clustering of viruses with identical sequences. This sustained transmission of MeVs in different regions of the Philippines often caused a spillover and it is associated with MeV importation to other countries. In Japan and the USA, imported cases of D9 MeV from the Philippines were documented and reported in 2010 and 2011 [9–11].

Another limitation of this study is the lack of historical support for tMRCA and mutation rate estimates. Comparison of our estimates to previously reported mean substitution rates showed similar results with MeVs in Spain, China, and other countries [12–15]. Our estimates were relatively lower but overlapped with the mutation rates of other RNA viruses such as human metapneumovirus [24] and influenza A virus [25]. This suggests that MeVs, like most RNA viruses exhibit high mutation rates, short generation times, and large population sizes due to its high reproductive number [24].

Genotype D9 measles virus was not the only strain that was circulating in the Philippines from 2008 to 2011. Sporadic cases of genotype G3 MeVs were detected from July 2008 to March 2010. Genotype G3 was reported first in 1999 in Australia, where this genotype was associated with local outbreaks and importation from East Timor [26]. In 2002, genotype G3 was isolated in East Timor and Indonesia, where this genotype was found to be endemic [27]. In the Philippines, genotype G3 was detected first in 2007 and cocirculated with genotype D9 [8]. While genotype D9 was detected in all regions in the country, genotype G3 circulation was limited only in Mindanao in late 2008 and in Panay Island in 2009 and 2010. Genotypes D9 and G3 cocirculated in Panay Island in 2010 but only D9 was detected in the following year. Phylogenetic and tMRCA analyses suggest that genotype G3 MeV might be imported from East Timor and Indonesia, where G3 and D9 MeVs are endemic [16, 26]. Continued surveillance must be performed to determine if the G3 genotype would reemerge in the Philippines.

## Conclusion

Genetic analysis showed that genotype D9 was responsible for the large measles outbreaks in the Philippines in 2010 and 2011. Genetic analysis revealed that genotype D9 virus became endemic in the country. Genotype G3 MeV was detected sporadically and its circulation occurred only in limited geographic parts of the country. This study highlights the importance of genetic analysis in helping with the assessment of measles elimination program in the country. This study underscores the need for continued high measles vaccination coverage to minimize the number of susceptible population, stop the transmission of endemic MeVs, prevent importation of MeVs in other countries, and meet the goal of measles elimination in the Philippines. This study provides important genetic surveillance data that can help monitor the progress of measles elimination in the country and help establish the transmission route of MeVs in the region and other parts of the world.

## Methods

### Clinical samples

A total of 565 serum samples that were tested positive for measles-specific IgM antibody were selected in this study. These samples were collected from suspected measles cases from different parts of the country and were sent to RITM for ELISA-based testing for anti-measles IgM antibodies [7]. At least one sample per month and per region was randomly selected from July 2008 to November 2011. Only samples that were collected within 3 days from onset of rash were included in this study. This study was approved by the Ethics Committee of Tohoku University Graduate School of Medicine and the Institutional Review Board (IRB) of RITM. Written informed consent was obtained from patients or patients’ parents or guardians before samples were collected.

### RNA extraction and RT-PCR

Total RNA was extracted directly from serum samples using the PureLink Viral RNA/DNA MiniKit (Invitrogen, Carlsbad, CA). RT-PCR was performed using One-Step RT-PCR kit (Qiagen, Hilden, Germany). Gene-specific primers were utilized to amplify the C-terminal region of the N gene (456 nt) and partial H gene (309 nt) using conventional PCR and nested PCR protocols as described previously [28, 29].

### Sequencing and phylogenetic analyses

PCR products were purified using SUPRIC^TM^-PCR (TaKaRa, Otsu, Japan) kit. Direct sequencing of PCR product was performed using BigDye Terminator v3.1 Cycle Sequencing Kit (Applied Biosystems, Foster City, CA, USA). Sequencing products were analyzed using the ABI Genetic Analyzer 3130 (Applied Biosystems, Foster City, CA, USA).

Phylogenetic analysis was performed using the neighbor-joining (NJ) method in Molecular Evolution Genetic Analysis (MEGA) software 5.0 [30]. Statistical support was tested using the bootstrap method with 1,000 replicates and values >60% were indicated on the branches. Sequences of reference strains for each genotype and sequences from other countries were downloaded from GenBank and were included in the phylogenetic analysis. Pairwise p-distance for each measles sequence was calculated using MEGA software 5.0.

To estimate the rate of evolution and the time to the most recent common ancestor (tMRCA) of MeV in the Philippines, a Bayesian Markov chain Monte Carlo (MCMC) method was used as implemented in the BEAST package 1.8.0 [31, 32]. For tMRCA analysis, only samples from the Philippines were included in the dataset. Sample collection dates were incorporated in the N gene sequence dataset to calibrate the molecular clock [33]. The best-fitting nucleotide substitution model was determined using the MEGA software 5.0 [30]. Based on the smallest Akaike information criterion (AIC) value, the Hasegawa–Kishino–Yano (HKY) model was selected as the best-fitting model [34]. The dataset was analyzed in BEAST using two different clock models (strict and uncorrelated lognormal relaxed) and two different demographic models (constant and exponential population growth) [35]. Results were compared to ensure that convergence was attained in each run and statistical summaries were combined after removing 10% as burn-in using the Tracer software in the BEAST package [31]. The best-fitting model for the dataset was determined by computing the Bayes factors using the Tracer software. Statistical uncertainties in the estimates were indicated by the 95% highest posterior density (HPD) intervals. Maximum clade credibility (MCC) trees were inferred also using the same N and H datasets in the NJ method for comparison of clustering of the Philippine samples with reference strains. Posterior probability values were indicated on the node of MCC trees. To estimate the genetic diversity of MeV overtime, a coalescent-based Bayesian skyline analysis was performed using the identified best-fitting clock and demographic models. Sequences described in this study were submitted to GenBank (N gene: KM066693-KM066717; H gene: KM 066718-KM066816).

## Additional files

Additional file 1:
**Figure S1.** Maximum clade credibility (MCC) tree of the N gene sequence of measles virus in the Philippines using the Bayesian Markov chain Monte Carlo (MCMC) method. X-axis represents the year of virus detection or isolation. Samples that were detected in 2008 are indicated by blue font color; 2009 (green); 2010 (orange); and 2011 (red). Genotype D9 MeVs are highlighted in blue box and genotype G3 viruses are highlighted in green box. Figures near the tree nodes represent posterior probability values. The scale bar represents nucleotide substitutions per site per year. Additional file 2:
**Table** **S1.**
Additional file 3:
**Figure S2.** Maximum clade credibility (MCC) tree of the partial H gene sequence of measles virus in the Philippines using the Bayesian Markov chain Monte Carlo (MCMC) method. X-axis represents the year of virus detection or isolation. Samples that were detected in 2008 are indicated by blue font color; 2009 (green); 2010 (orange); and 2011 (red). Genotype D9 MeVs are highlighted in blue box and genotype G3 viruses are highlighted in yellow-green box. Figures near the tree nodes represent posterior probability values. The scale bar represents nucleotide substitutions per site per year. Additional file 4:
**Table S2.**
Additional file 5:
**Figure S3.** Bayesian skyline plot of the H gene of genotype D9 MeVs in the Philippines. The estimate of the relative genetic diversity is plotted as a function of sample collection date. Thick black line represents the median value and light blue lines represent the high and low 95% highest posterior densities (HPD).

## References

[1] Moss WJ, Griffin DE (2012). Measles. Lancet.

[2] Knipe DM, Howley PM (2013). Fields virology.

[3] Mulders MN, Truong AT, Muller CP (2001). Monitoring of measles elimination using molecular epidemiology. Vaccine.

[4] WHO (2012). WHO: Measles virus nomenclature update: 2012. Wkly Epidemiol Rec.

[5] WHO (2013) Country profile for measles elimination: Philippines. WHO Western Pacific Region

[6] Sobel H, Ducusin J, De Quiroz M, Cabotaje M, Olive JM (2009). The Philippines 2004 measles campaign: a success story towards elimination. Trop Doct.

[7] WHO (2007) Manual for the laboratory diagnosis of measles and rubella virus infection, 2nd edn. World Health Organization

[8] Fuji N, Suzuki A, Saito M, Centeno R, Galang H, Lupisan S (2011). Interruption of the circulation of an indigenous measles genotype and the introduction of other genotypes after a mass vaccination campaign in the Philippines. J Med Virol.

[9] National Institute of Infectious Diseases (2010) Infectious agents surveillance report. In: Book infectious agents surveillance report, 2010, Oct edition

[10] National Institute of Infectious Diseases (2011) Infectious agents surveillance report. In: Book infectious agents surveillance report, 2011, Jan edition

[11] Centers for Disease C, Prevention (2012). Measles—United States, 2011. MMWR Morb Mortal Wkly Rep.

[12] Rima BK, Earle JA, Baczko K, ter Meulen V, Liebert UG, Carstens C (1997). Sequence divergence of measles virus haemagglutinin during natural evolution and adaptation to cell culture. J Gen Virol.

[13] Munoz-Alia MA, Fernandez-Munoz R, Casasnovas JM, Porras-Mansilla R, Serrano-Pardo A, Pagan I (2014). Measles virus genetic evolution throughout an imported epidemic outbreak in a highly vaccinated population. Virus Res.

[14] Xu S, Zhang Y, Rivailler P, Wang H, Ji Y, Zhen Z (2014). Evolutionary genetics of genotype H1 measles viruses in China from 1993 to 2012. J Gen Virol.

[15] Pomeroy LW, Bjornstad ON, Holmes EC (2008). The evolutionary and epidemiological dynamics of the paramyxoviridae. J Mol Evol.

[16] Rota PA, Brown K, Mankertz A, Santibanez S, Shulga S, Muller CP (2011). Global distribution of measles genotypes and measles molecular epidemiology. J Infect Dis.

[17] Chibo D, Riddell M, Catton M, Lyon M, Lum G, Birch C (2003). Studies of measles viruses circulating in Australia between 1999 and 2001 reveals a new genotype. Virus Res.

[18] Centers for Disease C, Prevention (2002). Outbreak of measles–Venezuela and Colombia, 2001–2002. MMWR Morb Mortal Wkly Rep.

[19] Mizuta K, Abiko C, Murata T, Yamada K, Ahiko T, Sakamoto M (2005). An outbreak of measles virus infection due to a genotype D9 at a junior high school in Yamagata, Japan in 2004. Jpn J Infect Dis.

[20] Riddell MA, Rota JS, Rota PA (2005). Review of the temporal and geographical distribution of measles virus genotypes in the prevaccine and postvaccine eras. Virol J.

[21] Cheng WY, Tung HP, Wang HC, Lee LL, Wu HS, Liu MT (2013). Molecular epidemiology of measles virus in Taiwan in 2010–2011: the common genotype changed from H1 to D9 and the first appearance of D4. J Med Virol.

[22] Pattamadilok S, Incomserb P, Primsirikunawut A, Lukebua A, Rota PA, Sawanpanyalert P (2012). Genetic characterization of measles viruses that circulated in Thailand from 1998 to 2008. J Med Virol.

[23] Cheng WY, Lee L, Rota PA, Yang DC (2009). Molecular evolution of measles viruses circulated in Taiwan 1992–2008. Virol J.

[24] de Graaf M, Osterhaus AD, Fouchier RA, Holmes EC (2008). Evolutionary dynamics of human and avian metapneumoviruses. J Gen Virol.

[25] Smith G, Vijaykrishna D, Bahl J, Lycett S, Worobey M, Pybus O (2009). Origins and evolutionary genomics of the 2009 swine-origin H1N1 influenza A epidemic. Nature.

[26] Chibo D, Riddell M, Catton M, Birch C (2002). Novel measles virus genotype, East Timor and Australia. Emerg Infect Dis.

[27] Rota PA, Bellini WJ (2003). Update on the global distribution of genotypes of wild type measles viruses. J Infect Dis.

[28] National Institute of Infectious Diseases (2003) Pathogen detection manual

[29] Santibanez S, Tischer A, Heider A, Siedler A, Hengel H (2002). Rapid replacement of endemic measles virus genotypes. J Gen Virol.

[30] Tamura K, Peterson D, Peterson N, Stecher G, Nei M, Kumar S (2011). MEGA5: molecular evolutionary genetics analysis using maximum likelihood, evolutionary distance, and maximum parsimony methods. Mol Biol Evol.

[31] Drummond AJ, Rambaut A (2007). BEAST: Bayesian evolutionary analysis by sampling trees. BMC Evol Biol.

[32] Drummond AJ, Nicholls GK, Rodrigo AG, Solomon W (2002). Estimating mutation parameters, population history and genealogy simultaneously from temporally spaced sequence data. Genetics.

[33] Rambaut A (2000). Estimating the rate of molecular evolution: incorporating non-contemporaneous sequences into maximum likelihood phylogenies. Bioinformatics.

[34] Hasegawa M, Kishino H, Yano T (1985). Dating of the human-ape splitting by a molecular clock of mitochondrial DNA. J Mol Evol.

[35] Drummond AJ, Ho SY, Phillips MJ, Rambaut A (2006). Relaxed phylogenetics and dating with confidence. PLoS Biol.

