# Supplementary figures and images for: Genetic characterization of measles virus in the Philippines, 2008–2011
Source: BMC Res Notes. 2015 Jun 3;8:211. doi: 10.1186/s13104-015-1201-1 (PMC4467837; doi:10.1186/s13104-015-1201-1)

## Slide 1
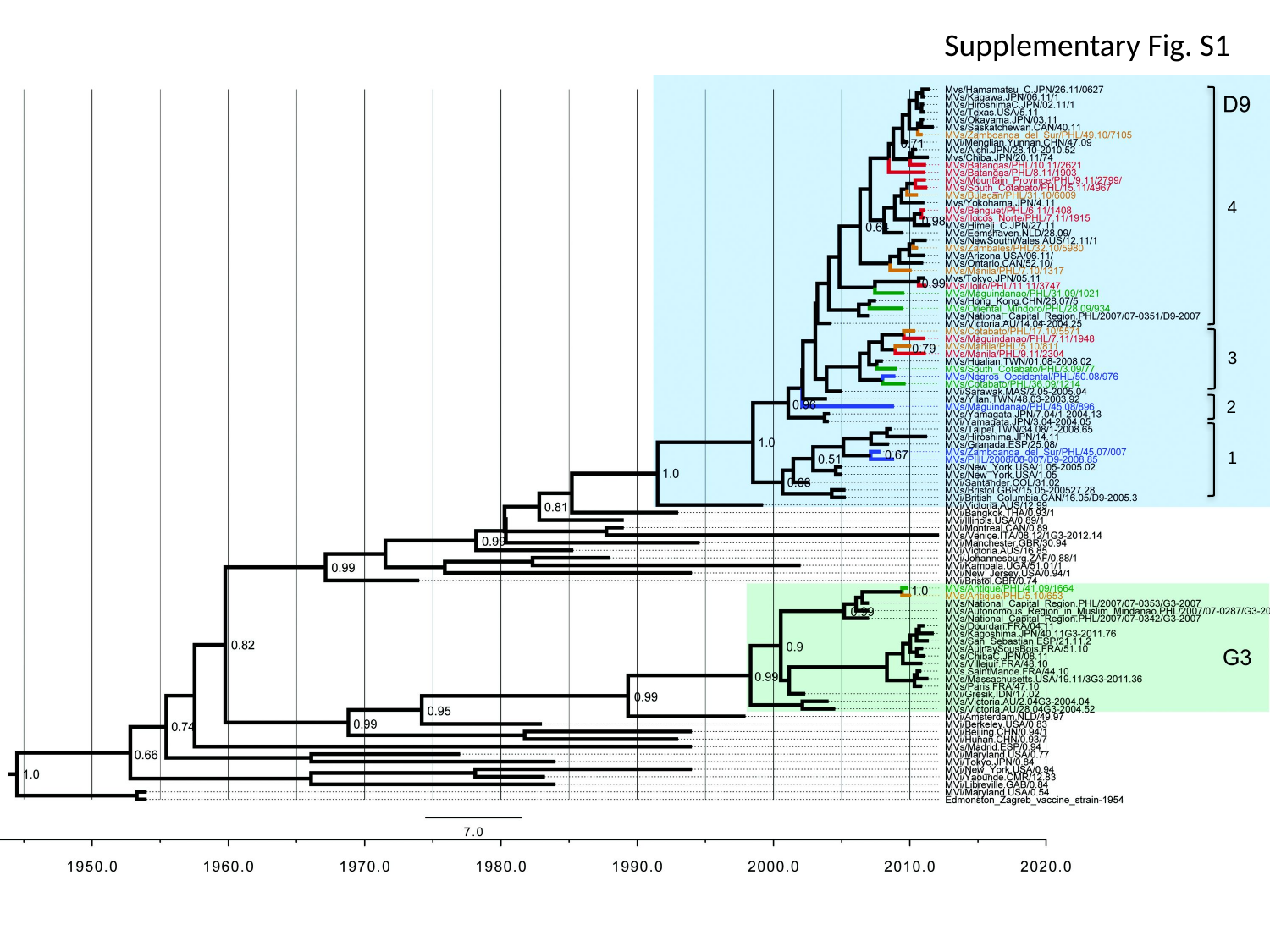

Supplementary Fig. S1
4
3
2
1

Supplement: Additional file 1: — Figure S1. Maximum clade credibility (MCC) tree of the N gene sequence of measles virus in the Philippines using the Bayesian Markov chain Monte Carlo (MCMC) method. X-axis represents the year of virus detection or isolation. Samples that were detected in 2008 are indicated by blue font color; 2009 (green); 2010 (orange); and 2011 (red). Genotype D9 MeVs are highlighted in blue box and genotype G3 viruses are highlighted in green box. Figures near the tree nodes represent posterior probability values. The scale bar represents nucleotide substitutions per site per year. [file 13104_2015_1201_MOESM1_ESM.pptx]

## Slide 1
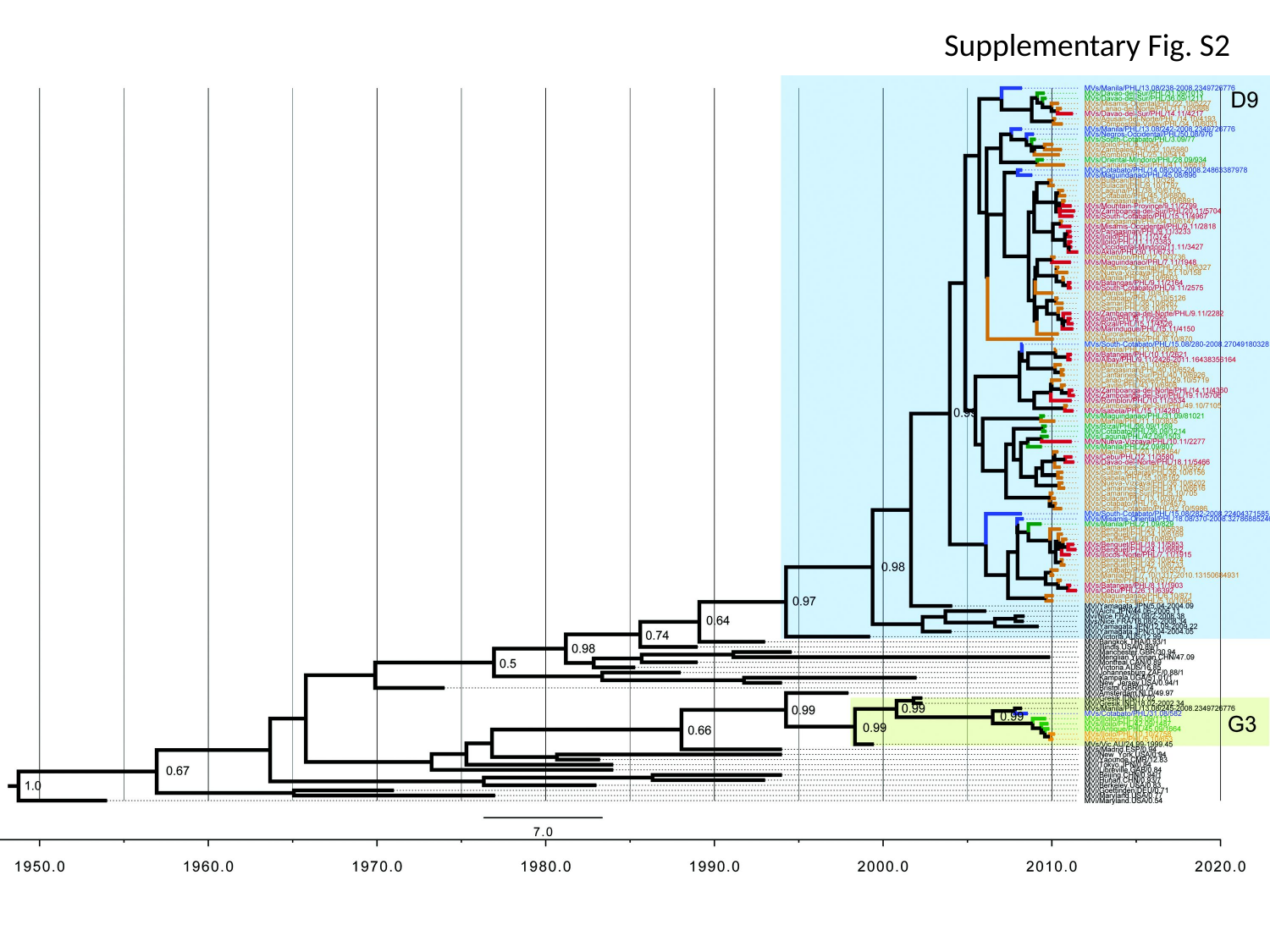

Supplementary Fig. S2

Supplement: Additional file 3: — Figure S2. Maximum clade credibility (MCC) tree of the partial H gene sequence of measles virus in the Philippines using the Bayesian Markov chain Monte Carlo (MCMC) method. X-axis represents the year of virus detection or isolation. Samples that were detected in 2008 are indicated by blue font color; 2009 (green); 2010 (orange); and 2011 (red). Genotype D9 MeVs are highlighted in blue box and genotype G3 viruses are highlighted in yellow-green box. Figures near the tree nodes represent posterior probability values. The scale bar represents nucleotide substitutions per site per year. [file 13104_2015_1201_MOESM3_ESM.pptx]

## Slide 1
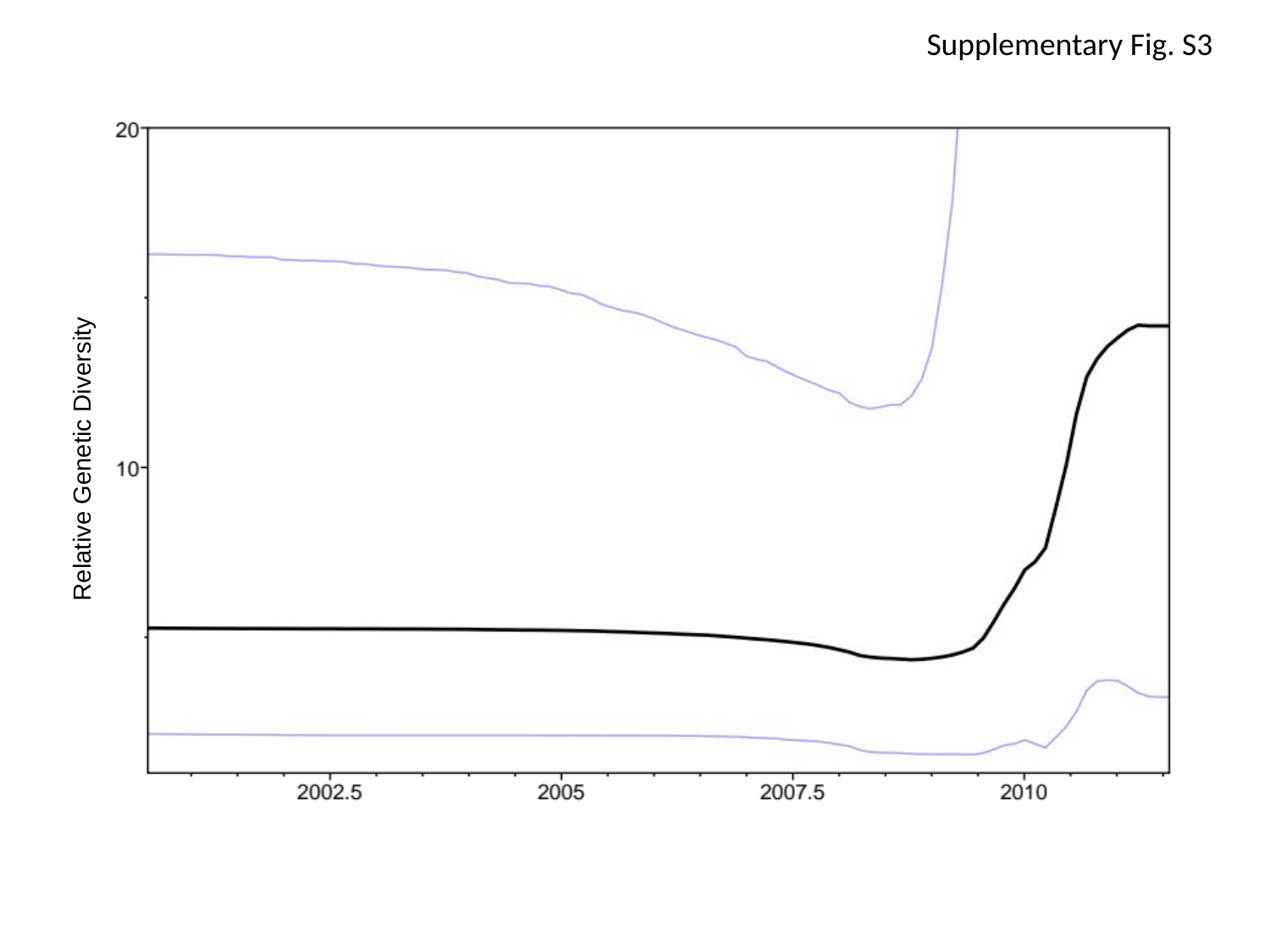

Supplementary Fig. S3
Relative Genetic Diversity

Supplement: Additional file 5: — Figure S3. Bayesian skyline plot of the H gene of genotype D9 MeVs in the Philippines. The estimate of the relative genetic diversity is plotted as a function of sample collection date. Thick black line represents the median value and light blue lines represent the high and low 95% highest posterior densities (HPD). [file 13104_2015_1201_MOESM5_ESM.pptx]
